# Meteorological Table

**Published:** 1860-12

**Authors:** 


### Register of Meteorological Observations,

Taken for Atlanta Medical and Surgical Journal, November, 1860, at Atlanta, Ga. Latitude 33 deg. 45 min.—Longitude 7 deg. 30 min.—Height above the Sea, 1050 feet.

| Days of Month | BAROMETER. | | | THERMOMETER. | | | RAIN AND SNOW. | | CLOUDS. | | | WIND. | | |
|---|---|---|---|---|---|---|---|---|---|---|---|---|---|---|
| | 7 A.M. | 2 P.M. | 9 P.M. | 7 A.M. | 2 P.M. | 9 P.M. | Amt. Rn | In. Sn'w | 7 A.M. | 2 P.M. | 9 P.M. | 7 A.M. | 2 P.M. | 9 P.M. |
| 1 | 28.70 | 28.60 | 28.60 | 58 | 64 | 40 | 1.50 | | 10 | 10 | 10 | S E | S E | S |
| 2 | 28.65 | 28.70 | 28.70 | 36 | 52 | 42 | | | 0 | 0 | 0 | N W | N W | N W |
| 3 | 28.70 | 28.75 | 28.75 | 40 | 64 | 50 | | | 0 | 0 | 0 | N W | N W | N W |
| 4 | 28.80 | 28.80 | 28.75 | 36 | 58 | 40 | | | 0 | 0 | 0 | N W | N W | N W |
| 5 | 28.70 | 28.75 | 28.70 | 36 | 64 | 50 | | | 0 | 5 | 5 | N W | N W | N |
| 6 | 28.70 | 28.70 | 28.75 | 46 | 58 | 48 | | | 5 | 0 | 0 | N | N | N |
| 7 | 28.75 | 28.75 | 28.80 | 34 | 50 | 40 | | | 0 | 0 | 5 | N E | N E | N E |
| 8 | 28.80 | 28.75 | 28.70 | 44 | 50 | 50 | | | 10 | 10 | 10 | N E | E | E |
| 9 | 28.45 | 28.66 | 28.60 | 50 | 50 | 40 | .50 | | 10 | 5 | 0 | S W | S W | S W |
| 10 | 28.65 | 28.70 | 28.70 | 34 | 50 | 44 | | | 0 | 0 | 0 | N W | N W | N W |
| 11 | 28.65 | 28.75 | 28.65 | 40 | 60 | 53 | | | 0 | 0 | 0 | N W | N W | N W |
| 12 | 28.65 | 28.70 | 28.70 | 49 | 68 | 55 | | | 0 | 0 | 0 | N W | N W | N W |
| 13 | 28.70 | 28.80 | 28.80 | 50 | 68 | 56 | | | 0 | 5 | 0 | N W | N W | N W |
| 14 | 29.00 | 29.00 | 29.00 | 48 | 58 | 50 | | | 0 | 5 | 0 | N W | N W | N W |
| 15 | 29.00 | 28.90 | 28.80 | 50 | 60 | 60 | | | 0 | 5 | 10 | N W | N | S W |
| 16 | 28.60 | 28.65 | 28.80 | 48 | 44 | 40 | .75 | | 10 | 10 | 10 | S W | S W | S W |
| 17 | 28.70 | 28.68 | 28.70 | 48 | 54 | 40 | | | 10 | 5 | 0 | N W | N W | N W |
| 18 | 28.70 | 28.68 | 28.70 | 48 | 56 | 46 | | | 10 | 5 | 0 | N W | N W | N W |
| 19 | 28.70 | 28.60 | 28.70 | 50 | 60 | 46 | | | 5 | 0 | 5 | W | W | W |
| 20 | 28.80 | 28.85 | 28.90 | 46 | 54 | 46 | | | 0 | 0 | 0 | W | W | W |
| 21 | 28.80 | 28.80 | 28.80 | 40 | 50 | 40 | | | 0 | 0 | 5 | N | N E | E |
| 22 | 28.80 | 29.00 | 28.80 | 44 | 54 | 40 | .50 | | 10 | 10 | 5 | E | W | N W |
| 23 | 28.80 | 29.00 | 29.00 | 36 | 44 | 32 | | | 0 | 0 | 0 | N W | N W | N W |
| 24 | 28.80 | 28.90 | 29.00 | 32 | 36 | 30 | | | 0 | 0 | 0 | N W | N W | N W |
| 25 | 29.00 | 29.10 | 29.20 | 24 | 32 | 30 | | | 5 | 5 | 10 | N E | N E | S E |
| 26 | 29.20 | 29.10 | 29.05 | 34 | 52 | 50 | | | 10 | 10 | 10 | S E | S E | W |
| 27 | 29.00 | 29.00 | 28.90 | 48 | 56 | 50 | 2.00 | | 10 | 10 | 10 | W | W | W |
| 28 | 28.85 | 28.80 | 28.80 | 52 | 58 | 50 | | | 5 | 0 | 0 | W | W | N W |
| 29 | 28.80 | 28.90 | 28.90 | 50 | 58 | 48 | | | 0 | 0 | 0 | N W | N W | N W |
| 30 | 28.75 | 28.70 | 28.80 | 42 | 58 | 38 | | | 0 | 0 | 0 | N W | N W | N W |

Explanation of Table.—0 Signifies Entire Clearness—5 Half Cloudy—10 Entire Cloudiness.

